# Imaging-based patient-reported outcomes (PROs) database: How we do it

**DOI:** 10.1007/s00256-020-03602-w

**Published:** 2020-09-18

**Authors:** Soterios Gyftopoulos, Adam Jacobs, Mohammad Samim

**Affiliations:** 1 Department of Radiology, NYU Langone Health, 660 First Avenue, New York, NY 10016 USA; 2 Department of Orthopedic Surgery, NYU Langone Health, 301 East 17th Street, New York, NY 10003 USA

**Keywords:** Anterior shoulder instability, Patient-reported outcomes, PROMs, Outcomes research, Radiology

## Abstract

Patient-reported outcomes (PROs) provide an essential understanding of the impact a condition or treatment has on a patient, while complementing other, more traditional outcomes information like survival and time to symptom resolution. PROs have become increasingly important in medicine with the push toward patient-centered care. The creation of a PROs database within an institution or practice provides a way to collect, understand, and use this kind of patient feedback to inform quality improvement and develop the evidence base for medical decision-making and on a larger scale could potentially help determine national standards of care and treatment guidelines. This paper provides a first-hand account of our experience setting up an imaging-based PROs database at our institution and is organized into steps the reader can follow for creating a PROs database of their own. Given the limited use of PROs within both diagnostic and interventional radiology, we hope our paper stimulates a new interest among radiologists who may have never considered outcomes work in the past.

## Introduction

Traditionally, radiology outcomes have consisted of measures of accuracy, image quality, and turnaround times. With medicine’s increasing push toward patient-centered care, collecting, understanding, and using patient feedback to improve management are becoming a top priority. A patient-reported outcome (PRO) is an observation that a patient provides about an aspect of their health state without interpretation by a medical professional [1]. PROs provide an understanding of the impact that a condition or treatment has on a patient’s life and their activities. PROs complement other outcome information, such as patient survival and time to symptom resolution, which have classically been used to assess a condition’s or treatment’s effect on a patient’s life [1–3]. A patient-reported outcome measure (PROM) is a tool used to collect and measure PROs [1]. They are typically created in survey format and filled out by the patient directly [1]. PROMs vary in scope, from general to disease-specific and, in terms of the types of data they collect, from information on the psychological impact of a disease to a condition’s physical symptoms and their impact on a patient’s daily life [2, 4].

The use of PROs in radiology as they relate to diagnostic imaging and image-guided interventions is lacking [5]. For diagnostic imaging, it can be difficult to measure an imaging study’s impact on a patient. From the interventional perspective, the limited number of PROMs for conditions that interventional radiologists treat and the heterogeneity of how interventional radiologists practice across the United States and globally make it difficult to standardize PROs collection and analysis [6]. In both groups, issues related to the need for administrative support to assist PROs collection and organization have also limited their use in our field [6]. While significant, these barriers to PROs utilization in radiology can be overcome with formal education and the sharing of experiences (both good and bad) by those experienced in outcomes work. Fortunately, there are many educational resources, including dedicated outcomes seminars, courses, journal articles, and consensus panel recommendations, which can provide information and guidance to radiologists interested in patient outcomes [6–12].

While the relevance of PROs to diagnostic imaging is less intuitive than it is to interventional procedures, many exciting areas of opportunity exist. PROs can help determine the most important aspects of diagnostic imaging for patient outcomes (potentially spanning the entire imaging experience from registration to the finalized report), a topic that has been incompletely explored up until this point [13]. They can also drive collaboration and improved communication between physicians of different specialties who treat a similar patient population, resulting in improved patient care. For radiologists working with clinicians, this could mean defining imaging findings that help make the best treatment decisions based on PROs. PROs can also help determine appropriate imaging by providing patient-specific information that could supplement (or potentially even replace) current markers of appropriateness such as congruence with established imaging algorithms.

PROs databases, containing information collected with PROMs, are crucial to the future of a patient-centered radiology practice seeking to determine, measure, and report the value it provides patients. The patient data collected in this format can inform both continuous quality improvement to improve processes and the evidence base to enhance patient and physician decisions when considering these interventions [14–16]. On a larger scale, PROs have the potential to help determine national standards of care and treatment guidelines for a variety of patient conditions, while providing increased opportunities for personalized medicine for radiologists [17]. While their importance is without question, creating a PROs database can be difficult.

The purpose of this paper is to provide a first-hand account of setting up an imaging-based PROs database. We will provide our experiences and lessons learned in terms of planning, developing, and operationalizing our database. Analysis of the collected data is beyond the scope of this paper. We hope this will be useful as a guide for radiologists interested in starting this type of work, whether it be for clinical or research purposes, while supplementing the resources currently available. We also hope this may stimulate a new interest among radiologists who have never considered outcomes work. We will use a collaborative musculoskeletal (MSK)/orthopedic surgery clinical research study using MRI that we are currently leading as a way to provide context to the information we are sharing. That being said, the lessons and experiences provided will be applicable to other types of diagnostic imaging as well as interventional radiology.

## What you need to create a PROs database

**Fig. 1:**
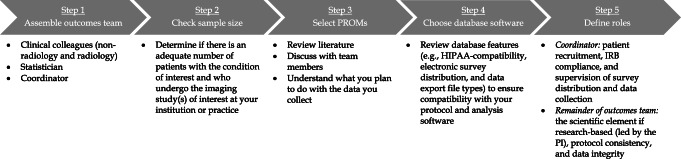
Five steps to create an imaging-based patient-reported outcomes (PROs) database. HIPAA Health Insurance Portability and Accountability Act, IRB institutional review board, PI principal investigator, PROMs patient-reported outcome measures

**Fig. 2:**
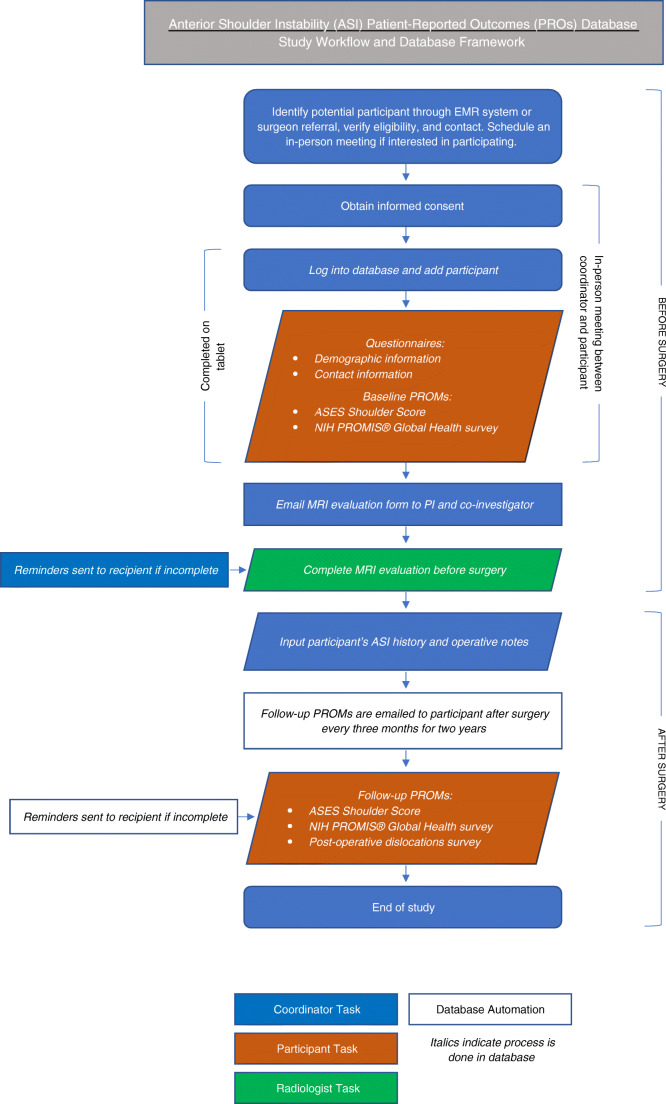
A simplified study workflow and database framework for our imaging-based PROs database. ASES American Shoulder and Elbow Surgeons, EMR electronic medical record, NIH National Institutes of Health, PI principal investigator, PROMs patient-reported outcome measures, PROMIS® Patient-Reported Outcomes Measurement Information System

There are several factors that need to be considered by the radiologist interested in creating a PROs database, which we will present over the next several paragraphs (Fig. 1). These are the key points that have worked for us and led to the successful creation and organization of our PROs database for anterior shoulder instability (ASI) patients. The goal of our ASI PROs database is to define the imaging findings on pre-operative MRI that can be used to predict how a patient will do and feel after Bankart surgery (Fig. 2). We plan on using this information to help define an appropriate imaging and treatment algorithm with our orthopedic colleagues for this patient population.

Along the way, we will also include lessons learned during this journey that will, hopefully, help you avoid potential pitfalls that could negatively impact your PROs database experiences.

### Step 1: Put together your outcomes team

First, the radiologist needs to have an outcomes team made up of colleagues who are interested in the disease of interest, a statistician who can help with planning and analysis, and a coordinator who can run and maintain the database. This team will provide the support system and expertise to guide the planning and organization of the database. Our research team included orthopedic surgeons and MSK radiologists who shared a common interest in ASI patients. The combination of radiologists and clinicians made sure that we understood our condition of interest from every angle, which was key for several reasons, most important of which was the selection of the PROs tools. Our statistician was involved in every step of our database setup. Our coordinator was the last addition to the team, but was the most important as this role has been vital to our database’s success (this will be evident to you the reader in later sections).

### Step 2: Adequate sample size

Before the database build, the radiologist needs to make sure she has enough data to create meaningful results. This means determining whether there is an adequate number of patients with the condition of interest and who undergo the imaging study(s) of interest at their institution or practice. This can be done with the help of a statistician and clinical colleagues who take care of this population. Power analyses are crucial in the planning of most research studies, but especially for outcomes work given the amount of time and effort needed. Working with your statistician during the early stages for this and other similar planning questions will allow you to avoid making mistakes that could negatively impact the creation of the database and/or limit the utility of the collected data. We highly recommend doing a quantitative analysis of your imaging and clinical data to accurately predict the likelihood of having enough information to move forward with the database.

With medical record digitization, collecting data has become much easier. Nonetheless, we had some difficulty obtaining these data. For our project, we used ICD (International Classification of Diseases) and CPT (Current Procedural Terminology) codes to see how many ASI patients were seen, imaged with MRI, and treated surgically in the past few years for future projections to see if creating an ASI-based dashboard was realistic at our institution. We provided these data to our statistician who determined the likelihood of performing a reasonable analysis for our outcomes of interest using a power analysis.

This took much more time than we originally set aside for this task. At first, we had difficulty identifying the best way to pull the data and which were the best codes to use in order to get an accurate representation of our potential patient population. It took time (with multiple failed attempts) to track down the right administrators in the orthopedic surgery department who had the access and tools needed for the data exportation. We also did not appreciate the amount of time needed to review, clean, and validate the collected data. It took our team between two and three weeks to convert our initial data pull into actionable data to produce our projections for the database. These initial extensively thorough efforts were essential, since they allowed us to eventually move forward with building the database.

### Step 3: Selection of the PROMs

There are a variety of PROM instruments [2, 4]. They can vary in length, from a single question to several questions, as well as in the types of data collected, from information on general health status to symptoms related to specific conditions. An example of a general instrument is the visual analog scale (VAS), a tool used to measure a patient’s pain (acute and chronic) [18]. An example of a condition-specific instrument is the Knee Injury and Osteoarthritis Outcome Score (KOOS), which assesses a patient’s symptoms after knee injury [19]. Additionally, collecting PROMs regularly allows for monitoring changes in outcomes over time in a scientific and validated way, potentially providing invaluable data. A more granular discussion of the different types is beyond the scope of this paper, but it is important to be aware that you will likely have several options to choose from, especially if you are studying a broad clinical condition. The selection of the tool(s) will depend on a number of factors, but primarily the outcome information that will help answer your clinical or research question(s). A thorough review of the literature and discussions with your team members will be helpful for this selection. An understanding of what you plan to do with the collected information is also important. For example, if you would like to use your PROs data for a cost-effectiveness study, then selecting PROs that can be converted to utilities would be useful.

For our study, we wanted something shoulder-specific that could be used to monitor ASI’s impact on shoulder function in daily life, as well as a broader tool to assess general physical and mental health. We wanted a complete understanding of ASI’s impact on patients. After reviewing our options and project goals with our team, we selected the American Shoulder and Elbow Surgeons (ASES) Shoulder Score for the specific tool and the National Institutes of Health (NIH) PROMIS® (Patient-Reported Outcomes Measurement Information System) Global Health survey for the general instrument, which could also be used to create utilities for a future cost-effectiveness study.

### Step 4: Database technology: What do you pick?

There are certain factors you should consider when evaluating different options available to create your database. First, you want the database to be digital and HIPAA (Health Insurance Portability and Accountability Act) compatible. Ideally, you want it to distribute and collect your PROMs as well as store your data seamlessly. The days of paper surveys are hopefully over. You want your database to distribute your PROMs electronically via email for remote completion and/or on a device, such as a tablet, for in-person completion. You want the PROs data to be stored in the database in a format that is streamlined for routine review and endpoint analysis. You also want the data to be easily accessible for export in file types that are compatible with your review and analysis software (e.g., Microsoft Excel (Microsoft) or SPSS Statistics (IBM)). Database software with the degree of function and compatibility described here will make organizing and running your PROs database much easier than it would be otherwise.

We chose to use REDCap (Vanderbilt University) for our database because it had the characteristics that we found most important. It provided the seamless combination of PROMs distribution, collection, storage, and export that we wanted. REDCap is easy to use, with no prior experience or database expertise needed, and there are a variety of online resources available to help with any issues or questions that may arise.

For our ASI patients, baseline data, including pre-operative PROMs and MRI imaging findings, are added to the database before surgery. At different time points after surgery, patients receive our follow-up PROMs via automated emails directly from the database. The emails contain a link for the patients to complete the surveys online. Once a patient fills out their surveys and submits them, the data automatically populates our database. To ensure a high response rate, automated reminder emails are sent directly from the database to patients with incomplete surveys. Patients who still fail to complete the provided surveys receive a phone call from the study team. This phone call serves not only as a reminder for survey completion but also as an opportunity for the patient to have any additional questions they may have on the surveys and/or study answered by our team. This combined approach has been beneficial to our study, contributing to an overall survey response rate of 80%. We also use our REDCap database to house other patient information, including contact information and physical exam and operative notes, to have all potentially relevant information in one location.

We originally reached out to a data service at our institution, which works with researchers. Their fees to set up, organize, and run our database were estimated in the thousands of dollars. While we are sure they would have done an excellent job, it was too expensive for us. REDCap was free to use at our institution, which was a big plus as we could use our budget for other expenses, like patient reimbursements for taking part in our project.

There are other clinical data management software platforms available for the radiologist interested in an outcomes database (discussion of these options is beyond the scope of this paper). Our advice is to find one that combines everything you need seamlessly.

### Step 5: Defining roles

The most important tasks that need to be assigned are the ones related to patients, specifically recruitment and supervision of PROMs distribution and data collection. Given the importance of these activities, the key role will be the one of the coordinator. This person will need to own these tasks and make sure they are done correctly. It is up to the rest of the outcomes team to make sure the coordinator understands the purpose of the database in order to answer any potential questions from patients. The coordinator needs to fully understand the steps related to the survey distribution and how the data is collected to be able to problem-solve. The coordinator needs to understand and be compliant with their Institutional Review Board’s (IRB) policies, especially the informed consent process. The coordinator also needs to have a clear understanding of when someone else from the team needs to be involved in a patient- or database-related issue.

Other important tasks, such as protocol consistency and data-integrity assessment, can be shared among the remainder of the team. If you have a PROs database for research purposes, the scientific element (e.g., definition and evaluation of research question(s)) can be led by the principal investigator, but will be much more impactful if also shared with the team in order to make sure different perspectives and thoughts are accounted for at each step.

We initially struggled with defining the roles of the other team members. We wanted to find responsibilities that kept each member involved regularly in the project. We have found quality assurance tasks, such as reviewing our patients’ study imaging and surgeries to make sure they are consistent with our protocol, have been useful for keeping our team engaged while contributing to the overall quality of the database.

## Future directions

The goal of this paper is to share our experiences setting up an imaging-based PROs database. We have outlined the steps we took, and, so far, we have been successful in maintaining our database. While PROs databases are not new for fields such as orthopedic surgery and internal medicine, they remain relatively untapped for radiology. While we present what a PROs database could look like today, there is a very good chance that its look and operations will be different in the future. For example, new technologies that allow for easier PROs collection, such as text messaging survey questions to patients and having them text back answers, will increase patient response rates resulting in more, useful PROs data [20]. Additionally, artificial intelligence may provide easier ways to pull data from existing records in order to fill out PROMs without additional work by the patient or outcomes team. Even with these potential changes, the fundamentals will remain the same. Invest the time to set up your PROs database properly and you, your patients, and the field of radiology will benefit from this treatment-guiding information for years to come.
